# Multiflora rose invasion amplifies prevalence of Lyme disease pathogen, but not necessarily Lyme disease risk

**DOI:** 10.1186/s13071-018-2623-0

**Published:** 2018-01-23

**Authors:** Solny A. Adalsteinsson, W. Gregory Shriver, Andrias Hojgaard, Jacob L. Bowman, Dustin Brisson, Vincent D’Amico, Jeffrey J. Buler

**Affiliations:** 1 Department of Entomology and Wildlife Ecology, University of Delaware, Newark, DE USA; 2 Tyson Research Center, Washington University in St. Louis, St. Louis, MO USA; 3 Division of Vector-Borne Diseases, Centers for Disease Control and Prevention, Fort Collins, CO USA; 4 Department of Biology, University of Pennsylvania, Philadelphia, PA USA; 5 Northern Research Station, USDA Forest Service, Newark, DE USA

**Keywords:** Lyme disease, *Borrelia burgdorferi*, *Borrelia miyamotoi*, *Anaplasma phagocytophilum*, *Babesia microti*, Invasive species, Urbanization, Forest fragment

## Abstract

**Background:**

Forests in urban landscapes differ from their rural counterparts in ways that may alter vector-borne disease dynamics. In urban forest fragments, tick-borne pathogen prevalence is not well characterized; mitigating disease risk in densely-populated urban landscapes requires understanding ecological factors that affect pathogen prevalence. We trapped blacklegged tick (*Ixodes scapularis*) nymphs in urban forest fragments on the East Coast of the United States and used multiplex real-time PCR assays to quantify the prevalence of four zoonotic, tick-borne pathogens. We used Bayesian logistic regression and WAIC model selection to understand how vegetation, habitat, and landscape features of urban forests relate to the prevalence of *B. burgdorferi* (the causative agent of Lyme disease) among blacklegged ticks.

**Results:**

In the 258 nymphs tested, we detected *Borrelia burgdorferi* (11.2% of ticks), *Borrelia miyamotoi* (0.8%) and *Anaplasma phagocytophilum* (1.9%), but we did not find *Babesia microti* (0%). Ticks collected from forests invaded by non-native multiflora rose (*Rosa multiflora*) had greater *B. burgdorferi* infection rates (mean = 15.9%) than ticks collected from uninvaded forests (mean = 7.9%). Overall, *B. burgdorferi* prevalence among ticks was positively related to habitat features (e.g. coarse woody debris and total understory cover) favorable for competent reservoir host species.

**Conclusions:**

Understory structure provided by non-native, invasive shrubs appears to aggregate ticks and reservoir hosts, increasing opportunities for pathogen transmission. However, when we consider pathogen prevalence among nymphs in context with relative abundance of questing nymphs, invasive plants do not necessarily increase disease risk. Although pathogen prevalence is greater among ticks in invaded forests, the probability of encountering an infected tick remains greater in uninvaded forests characterized by thick litter layers, sparse understories, and relatively greater questing tick abundance in urban landscapes.

**Electronic supplementary material:**

The online version of this article (10.1186/s13071-018-2623-0) contains supplementary material, which is available to authorized users.

## Background

Urbanization affects many aspects of vector-borne disease ecology [1]. In the case of tick-borne disease systems such as Lyme disease (caused by *Borrelia burgdorferi*) in forested ecosystems, urbanization alters habitat suitability for vectors (i.e. ticks), vertebrate hosts, and as a result, pathogens. Human development in the Lyme disease endemic, mid-Atlantic region of the United States reduces overall forest cover and average patch size while increasing the area of edge and impervious surface. Reduced forest patch size, in particular, results in predictable changes to host community composition that increase acarological risk in terms of nymphal infection prevalence and density of infected nymphs [2–5]. Yet in human-dominated landscapes, patch size may have a smaller or perhaps unpredictable influence on host community relative to other effects of urbanization on forested ecosystems. How ecological characteristics of urban forest fragments affect acarological risk has not been well explored.

Complex land use histories in human-dominated landscapes form networks of diverse, heterogeneous forest fragments. In the urban mid-Atlantic region, clear-cutting, intensive agriculture, and urban sprawl have created a variety of forest fragment types on a spectrum between remnants of mature (> 100 yr. old) forests and forest fragments that have regrown from fallow agricultural land set aside while surrounding areas were developed [6]. In the latter case, native tree species have competed with and grown alongside non-native species that were part of the agricultural landscape or subsequent development. As a result, regrown urban forest patches have closed canopies of mostly native trees with thick understories composed of non-native, invasive species [7]. These two extremes of urban forest fragment types both face serious ecological problems (e.g. loss of native understory or reduced regeneration), with implications for tick-borne disease risk.

Due to changes to below-ground processes and browsing pressure from high density white-tailed deer (*Odocoileus virginianus*) populations (in Delaware, recent county surveys estimate between 18 and 52 deer/km^2^ [8]), mature forests may have sparse or no woody understories and cannot replace many species of dead or dying trees [9]. Although they maintain a thick litter layer and low soil pH, which may help buffer mature forests from invasion by non-native plants [10], many native woody plants cannot regenerate. The thick litter layer maintained in these forests provides suitable habitat for blacklegged ticks (*Ixodes scapularis*), which are found in greater abundance in mature forests relative to other urbanized forest fragment types [11]. In contrast, forest fragments with significant non-native plant invasion in the understory have high densities of invasive earthworms and very little leaf litter [12–15], which constrains tick abundance [11, 16, 17]. However, the dense understory structure provided by invasive plants may aggregate immature ticks and infective hosts, potentially amplifying acarological risk in invaded forest fragments [18–21].

Recent studies have identified greater pathogen prevalence in ticks and reservoir hosts associated with invasive shrubs [18–20, 22]. However, because leaf litter loss, which constrains tick abundance, is also associated with non-native plant invasion, it is unclear how tick-borne disease risk differs in regrown, invaded forest fragments compared to mature, uninvaded fragments. To contribute to our understanding of tick-borne disease ecology in urbanized landscapes, we designed a study in urban forest fragments with three objectives: (i) characterize *B. burgdorferi* and emerging tick-borne pathogen prevalence among questing ticks; (ii) test for differences in pathogen prevalence between forests invaded by non-native understory plants and uninvaded forests; and (iii) determine which habitat and landscape features influence pathogen prevalence.

## Methods

### Study area and tick collection

**Fig. 1:**
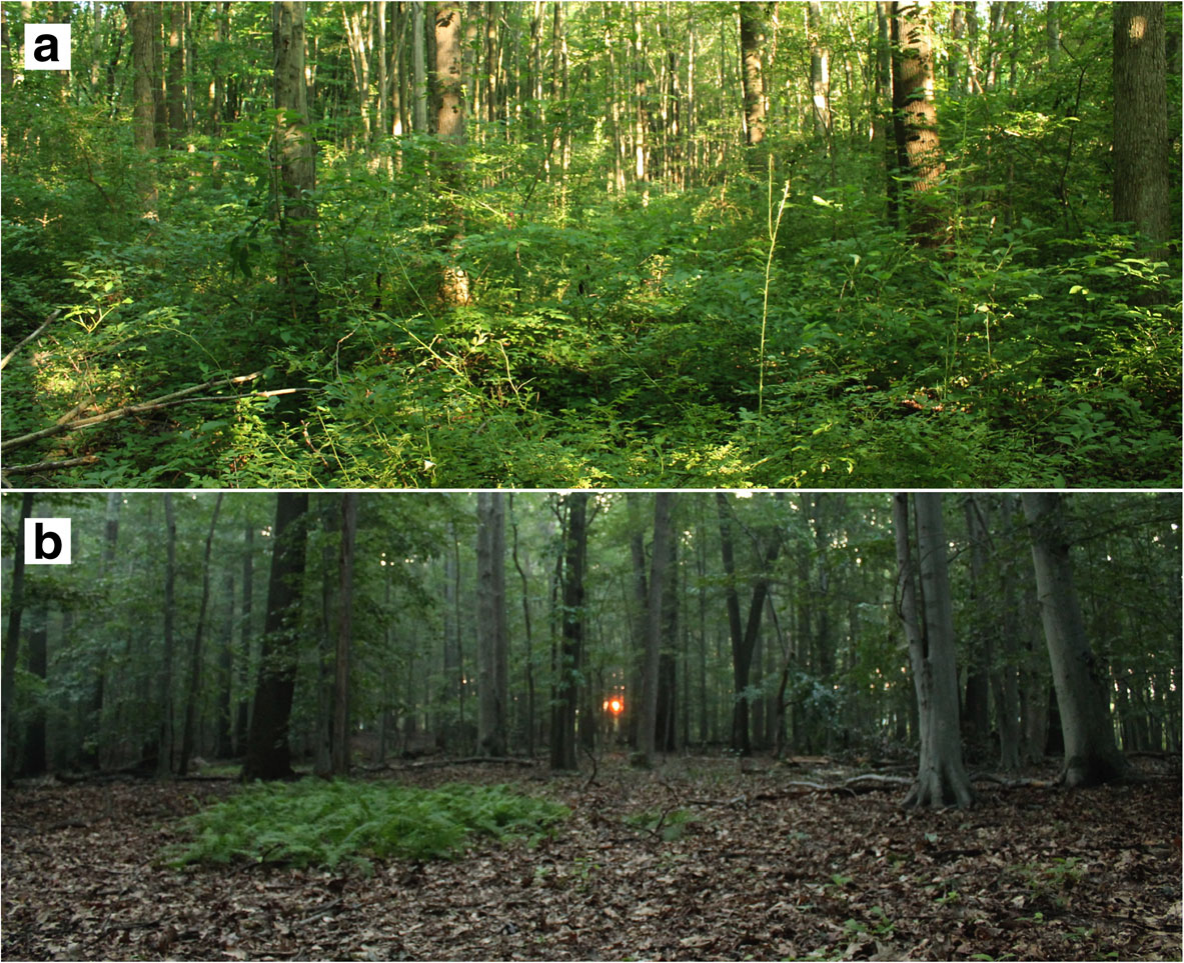
Photographs of representative forest sites with *R. multiflora* invasion (**a**) and without *R. multiflora* (**b**)

We collected nymphal *Ixodes scapularis* ticks from April to July, 2013 and 2014, using CO_2_-baited traps in forest fragments around New Castle County, Delaware. Although drag-sampling or flagging is more commonly used to capture *I. scapularis*, we used CO_2_-baited traps to avoid confounding results from *Rosa multiflora*’s dense structure (Fig. 1). Even thick, canvas cloth becomes snagged on *R. multiflora* thorns, preventing effective sampling of tick habitat. CO_2_-baited traps are unbiased by habitat structure [23]. We built traps following the design of Kensinger & Allan [23], by drilling four holes in 6-quart Coleman® coolers and bolting the coolers to plywood squares. We baited traps for 24 h with 1.4 kg of pelleted dry ice and lined the plywood base with doubled-sided carpet tape (3 M, Maplewood, USA).

Forest fragments (6–16 ha) consisted of mixed deciduous hardwood stands and varied in understory woody species composition, particularly in the extent of non-native *R. multiflora* invasion (Fig. 1, Additional file 1: Figure S1). Each year we trapped ticks in eight forest fragments, four of which had understories with 10–58% of total area covered by *R. multiflora* invasion (hereafter: invaded), and four fragments lacked *R. multiflora* invasion (< 1%), (hereafter: uninvaded). Within invaded sites, we captured ticks at four sets of paired traps: one trap within *R. multiflora* cover and its pair 25 m away, not in *R. multiflora*. Paired traps were separated by 25 m to eliminate the possibility that both traps could be attracting the same ticks [24]. In uninvaded sites, we deployed traps at four random points, separated by at least 25 m. We used a total of 64 trap locations over the 2 yr. study, and half of the traps were active on any given trap night. To avoid weather-related impacts on tick questing behavior, we always deployed paired traps together, and baited in equal numbers of invaded and uninvaded fragments on the same nights. We transported all captured ticks to the laboratory live, in individual microcentrifuge tubes, froze them at -80 °C, and later identified them to species and life stage with dichotomous keys [25–27].

### Covariate data collection

We identified a set of 25 variables that we expected would influence *B. burgdorferi* infection rates by increasing or decreasing interactions between larval ticks and competent reservoir hosts (Tables 1 and 2). Further detail on field and computational methods used to collect these data were published in [11, 28]. We surveyed understory vegetation characteristics within a 12.5 m radius surrounding each trap location, which included: estimating the percent of ground covered by *R. multiflora*, the percent of ground covered by coarse woody debris, and the density of understory vegetation using a 2.0 m high Nudds board for which observers estimated the percentage of each of four 0.5 m panels obscured by vegetation from a distance of 12.5 m [29]. We chose a 12.5 m radius to correspond with the approximate home range size of *Peromyscus leucopus* (an important reservoir for *B. burgdorferi*), while avoiding overlap with paired traps [30]. We also estimated the percent of ground covered by *R. multiflora* within a 2.5 m radius of the trap to more directly represent the effective trapping radius for *I. scapularis* [24]. We quantified leaf litter volume for all litter collected within a 0.5 m^2^ quadrat next to each trap.

**Table 1:** Summary of vegetation and landscape covariates measured at the trap scale by forest type and location, modified from [11]. Covariates are summarized as mean ± standard error. Different superscript letters A, B, C denote significant differences among groups (*P* < 0.05) detected using analysis of variance (ANOVA), blocking on site, followed up with Tukey’s *post-hoc* comparisons when there were more than two groups

Covariates	Uninvaded forests	Invaded: in rose	Invaded: not in rose
Trap-level covariates			
Nudds at 0.5–1.0 m (%)	18.0 ± 3.9^A^	73.9 ± 4.5^B^	53.3 ± 5.9^C^
Rose cover, 12.5 m radius (%)	2.5 ± 0.2^A^	11.2 ± 0.6^B^	7.0 ± 0.6^C^
Leaf litter volume (l/m^2^)	28.0 ± 2.8^A^	6.1 ± 1.4^B^	6.7 ± 1.2^B^
Coarse woody debris (%)	6.5 ± 0.9^A^	3.4 ± 0.7^B^	4.2 ± 0.7^B^
Rose cover, 2.5 m radius (%)	0.0 ± 0.0^A^	67.1 ± 2.2^B^	3.5 ± 0.7^A^
Distance to agriculture (m)	288.3 ± 54.7^A^	156.7 ± 24.6^B^	159.6 ± 24.6^B^
Distance to edge (m)	67.8 ± 9.1^A^	39.8 ± 8.8^B^	41.9 ± 8.4^B^
Distance to road (m)	154.7 ± 16.7	135 ± 18.1	133.4 ± 15.4
Distance to residential (m)	716.9 ± 377.6	186.2 ± 31.5	174.4 ± 32.9
Distance to stream (m)	371.8 ± 62.7^A^	148.4 ± 35.7^B^	134.1 ± 35.6^B^
Tick abundance^a^	0.8 ± 0.1^A^	0.4 ± 0.1^B^	0.2 ± 0.0^B^
Mouse abundance^b^	2.7 ± 0.5	4.5 ± 0.9	2.1 ± 0.4
Mean larvae per mouse^b^	0.4 ± 0.1	0.5 ± 0.1	0.7 ± 0.2

^a^Tick abundance values from traps that caught ticks which could be screened for pathogens [11]

^b^Mouse abundance and larval burdens on mice from concurrent nest box study (Adalsteinsson et al., unpublished data). For trap-level estimates, we calculated the mean of the mice caught during fall at two nest boxes in closest proximity to the tick trap. Larval burdens are the average number of larvae per mice at either the two closest nest boxes

“Nudds” refers to Nudds board (Nudds [29]) measurements and “dbh” stands for diameter at breast height

**Table 2:** Summary of vegetation and landscape covariates measured at the patch scale by forest type (invaded or uninvaded), modified from [11]. Covariates are summarized as mean ± standard error. Superscript letters A, B denote significant differences among groups (*P* < 0.05) detected using analysis of variance (ANOVA), blocking on site

Covariates	Uninvaded forests	Invaded forests
Patch-level covariates		
Rose cover (%)	0.8 ± 0.5^A^	36.9 ± 7.7^B^
Total understory cover (%)	19.6 ± 4.4^A^	41.6 ± 6.1^B^
Leaf litter volume (l/m^2^)	13.9 ± 1.1^A^	6.8 ± 0.9^B^
*Fagus grandifolia* (%)	8.5 ± 2.8^A^	0.7 ± 0.2^B^
*Acer* spp. (%)	0.7 ± 0.1^A^	21.2 ± 1.2^B^
Year of canopy closure	1916.7 ± 4.9^A^	1963 ± 5.1^B^
Non-native stems (%)	9.1 ± 2.7^A^	40.0 ± 3.3^B^
Average tree dbh (m)	0.6 ± 0.0	0.6 ± 0.0
*Quercus* spp. (%)	42.0 ± 6.4^A^	11.0 ± 5.8^B^
Mean mice per nest box^a^	0.4 ± 0.1	0.5 ± 0.2
Mean larvae per mouse^a^	0.7 ± 0.2	0.9 ± 0.3
Bird territory density^b^	3.6 ± 0.5	5.3 ± 0.8

^a^Mouse abundance and larval burdens on mice from concurrent nest box study (Adalsteinsson et al., unpublished data). Total mouse captures and larvae on mice during fall were averaged across all 15 nest boxes in the site

^b^Spot mapping data for all ground-foraging bird species, collected during 2010 and 2011 breeding seasons [28]

“dbh” stands for diameter at breast height

We measured landscape variables at each trap location in ArcGIS using a 2007 Delaware land use land cover layer [31], focusing on variables that could influence habitat suitability for ticks and/or hosts and that reflected the human-dominated landscape context of the study area [32, 33]: distance to nearest road, stream, agriculture, forest edge and residential development. We also used data from prior [11, 28] and concurrent studies (Adalsteinsson et al., unpublished data) to quantify abundance of ticks, potential hosts, and host-tick interactions in the study area. Tick abundance at the trap-level was the number of *I. scapularis* nymphs captured at a given trap, standardized by effort (number of trap nights). The densities of ground-foraging bird territories in forest fragments were estimated from spot-mapping surveys conducted during two breeding seasons [28]. Concurrent studies of *P. leucopus* abundance and parasitism by immature ticks (Adalsteinsson et al., unpublished data) provided estimates of mouse abundance and parasitism rates at the trap and forest fragment scale. To study *P. lecuopus* abundance, we checked 15 nest boxes per forest fragment once per month; for trap-level estimates, mouse abundance was the mean of the number of mice caught during fall (larval tick season) at the two nest boxes nearest to the trapping location. For patch-level estimates, the number of mice caught at nest boxes in fall was averaged across all 15 nest boxes in a given forest fragment. Larval tick burdens were the mean number of larvae per mouse at either the two closest nest boxes (trap-level) or across all 15 nest boxes (patch-level).

We also included data collected previously to characterize vegetation at the patch level: proportions of *Fagus grandifolia*, *Acer* spp., *Quercus* spp., *Liriodendron tulipifera*, or *Liquidambar styraciflua* as dominant canopy trees; percent of total area covered by *R. multiflora*; mean leaf litter volume measured at 15 locations in the patch; percent of ground covered by understory plants (all spp.); percentage of understory woody stems that were non-native; and year of canopy closure [28].

### Pathogen testing

We used a modified version of the DNeasy Blood & Tissue Kit (Qiagen, Venlo, Netherlands) protocol to extract DNA from ticks. Here, we explain the steps in which we deviated from the manufacturer’s protocol. First, we used sterile pipette tips to manually crush each *I. scapularis* nymph individually in 20 μl of Hyclone Dulbecco’s phosphate buffer saline solution (Thermo Fisher Scientific, Waltham, USA). Next, we incubated samples with lysis buffer ATL and proteinase K in a 56 °C hot water bath for 3 h. We performed an extra spin step at 13,000× *rpm* to remove trace ethanol after the Buffer AW2 wash. Finally, we modified the last step by eluting our samples twice (50 μl each time), for a final product of 100 μl. We checked concentrations of a subset of our samples using a NanoDrop UV-Vis spectrophotometer (Thermo Fisher Scientific, Waltham, USA) to confirm successful DNA extractions.

We tested ticks for the presence of *Borrelia burgdorferi* (*sensu lato*), *Anaplasma phagocytophilum*, and *Babesia microti* using a previously described multiplex PCR assay [34]. In addition, ticks were also tested for the presence of *Borrelia miyamotoi* in a TaqMan PCR assay using the following primers and probe: F770-5′-ACC TGC AAC CTT CGG ATT C-3′; R771-5′-TGG TTG TAG CTC AGT TGG TAG-3′; P1277-CalRd610-5′-CTT GTA TCG AAC TAC ACC CAT AGC TC-3′-BHQ2.

### Data analysis

A sufficient number of ticks tested positive for *Borrelia burgdorferi* to allow statistical analyses; however, infection prevalence was too low for the remaining pathogens to determine patterns related to invasion and other habitat and landscape features. We tested for spatial autocorrelation in *B. burgdorferi* prevalence across forest fragments using a spline correlogram in package *ncf* [35] in R [36].

Sample sizes were uneven because of differences in tick abundance in invaded and uninvaded sites [11], so we used logistic regression models in a Bayesian framework to compare pathogen (i.e. *B. burgdorferi*) prevalence among ticks collected from invaded and uninvaded forest fragments. This approach allowed us to include uncertainty in pathogen prevalence due to varying sample sizes rather than comparing raw proportions. We first tested whether pathogen prevalence differed among treatments (in rose vs not in rose) within invaded forest fragments. Next, we tested whether pathogen prevalence differed between invaded and uninvaded forests. For both of these questions, we used the following model structure:

$$ {\displaystyle \begin{array}{l}{Y}_i\sim \mathrm{Binomial}\left(1,{p}_i\right),\\ {}\mathrm{logit}\left({p}_i\right)=\alpha +{\beta}^{\ast }{X}_i,\end{array}} $$

where *Y* is the infection status of each individual (i) tick (0 or 1), which has a binomial distribution with probability *p*. With the logit link, the probability of infection (*p*) was estimated as a linear function of baseline infection probability (*α*) plus the effect of being from one of two treatment groups (β*X), where the variable *X* indicates whether the sampling site was either in rose or not in rose, or in an invaded forest or not (0 or 1), depending on the question. We specified vague priors for the parameters *α* and *β* including a normal distribution, and we used a Hamiltonian Monte Carlo sampler to run the analysis with STAN [37] using packages *rstan* [38] and *rethinking* [39] in R [36]. Using the difference between posterior distributions (89% highest posterior density intervals) of the estimated pathogen prevalence in two groups (in rose or not in rose, and invaded or uninvaded forest), we calculated the probability of infection prevalence in one treatment group being larger than the other, given our data and the specified model [40].

To understand the influence of habitat and landscape factors on *B. burgdorferi* prevalence, we used aggregated logistic regression models in a Bayesian framework [40]:

$$ {\displaystyle \begin{array}{l}{Y}_i\sim \mathrm{Binomial}\left(n,{p}_i\right),\\ {}\mathrm{logit}\left({p}_i\right)=\alpha +{\beta_1}^{\ast }{X}_{1i}+{\beta_2}^{\ast }{X}_{2i}+\dots +{\beta_z}^{\ast }{X}_{zi}.\end{array}} $$

Counts *of B. burgdorferi*-positive ticks (*Y*_*i*_) were aggregated by trap (*i*), and the number of trials (*n*) was the number of ticks tested from each trap. We developed multivariate model sets according to variable types (vegetation, habitat, or landscape) and spatial scale (trap or forest fragment). To select between models, we used Watanabe-Akaike Information Criterion (WAIC) [40–42] and converged on several “best models” that included similar combinations of variables (Table 3). We compared the relative influence of individual variables on the performance of the model by systematically dropping variables and comparing changes in WAIC scores and their standard errors [43].

**Table 3:** WAIC table of best models. Variables in the model set are coarse woody debris (CWD), leaf litter volume (litter), distance to the nearest road (dist. Road), mouse abundance in fall (mice), total understory cover (total cover), and nymphal tick capture rate (tick abundance). Field headings refer to the effective number of parameters (pWAIC), the difference between WAIC estimates for each model and the top-ranked model (ΔWAIC), the Akaike weight (Weight), the standard error of the WAIC estimate (SE), and the standard error of ΔWAIC value (ΔSE)

Model structure	ΔWAIC	pWAIC	Weight	SE	ΔSE
CWD + litter + dist. Road + mice	0.00	5.10	0.37	20.80	NA
CWD + litter + dist. Road	0.80	3.80	0.25	26.70	3.78
CWD + litter + dist. Road + mice + total cover	0.90	5.80	0.24	21.00	28.10
CWD + litter + dist. Road + mice + tick abundance	2.00	6.40	0.14	21.10	1.98
NULL	13.60	1.00	0.00	21.30	8.36

## Results

**Fig. 2:**
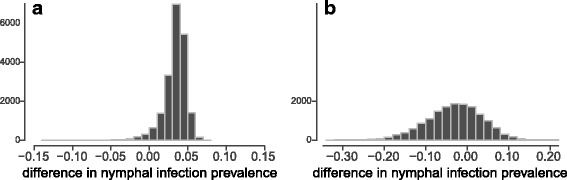
Distributions of differences between estimated *Borrelia burgdorferi* infection prevalence (posterior distributions) in nymphs between invaded and uninvaded forest fragments (**a**) and within invaded forests, between ticks captured within *Rosa multiflora* and outside of it (**b**). Y-axes display the density of samples from posterior distributions. Between invaded and uninvaded forests (**a**), there is 97% probability that *B. burgdorferi* prevalence is greater in invaded forests. Within invaded forests (**b**), there is only 64% probability that *B. burgdorferi* prevalence is lower within *R. multiflora* stands. Thus, we find support for a difference in *B. burgdorferi* prevalence at the forest-fragment scale (**a**), but not within invaded fragments (**b**)

We tested 258 *I. scapularis* nymphs, from which we successfully extracted DNA (determined through NanoDrop and PCR methods). Twenty-nine ticks (11.2%) were positive for *B. burgdorferi*, five (1.9%) were positive for *Anaplasma phagocytophilum*, and two (0.8%) were positive for *Borrelia miyamotoi.* Only one tick was co-infected with *B. burgdorferi* and *A. phagocytophilum.* We did not find any *Babesia microti*-positive ticks. Spatial autocorrelation in *B. burgdorferi* prevalence across forest fragments was not significant at any distance; at 0 m, the correlation coefficient was -0.61 (95% CI: -1.79–0.72). Within invaded forests, 11 of 75 ticks (14.6%) captured within *R. multiflora* and 6 of 32 ticks (18.7%) captured outside of *R. multiflora* were *B. burgdorferi-*positive. Of 151 ticks tested from uninvaded sites, 12 (7.9%) were positive for *B. burgdorferi*. Based on differences between posterior distributions, we estimated that there was a 64% probability that infection prevalence was greater outside of *R. multiflora* patches within invaded sites. However, there was a 97% probability that infection prevalence was higher among nymphs from invaded sites compared to nymphs from uninvaded sites (Fig. 2).

**Fig. 3:**
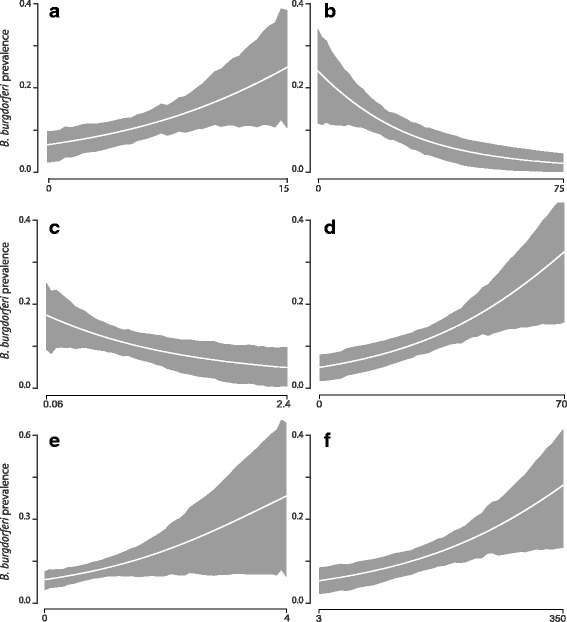
Mean model-averaged partial predicted responses (with 89% posterior probability intervals) of *Borrelia burgdorferi* prevalence among ticks (proportion of infected ticks) to six different variables: woody debris (%) (**a**); leaf litter volume (L/m^2^) (**b**); tick abundance (nymphs/24 h) (**c**); understory cover (%) (**d**); mouse abundance (mice per nest box per check) (**e**); distance to nearest road (m) (**f**). Overall, *B. burgdorferi* prevalence is predicted to increase with increasing woody debris, understory cover, mouse abundance, and distance to nearest road, while increasing leaf litter volume and tick abundance should decrease *B. burgdorferi* prevalence among ticks

Among the 50 models tested, the best models for *B. burgdorferi* infection prevalence (Table 3) included woody debris, leaf litter, distance to road, mouse abundance, tick abundance (all at trap-scale), and total cover (fragment-scale). Woody debris, distance to road, mouse abundance, and total cover were positively related to infection prevalence. Leaf litter and tick abundance were negatively related to infection prevalence (Fig. 3).

## Discussion

In our comparison of invaded and uninvaded forest fragments, we found that *B. burgdorferi* prevalence among questing ticks did not differ within invaded forests, but that the infection prevalence in ticks from invaded forests was almost double that in ticks from uninvaded forests. *Borrelia burgdorferi* was the most common pathogen detected in nymphal *I. scapularis* from our study sites, followed by *A. phagocytophilum* and *B. miyamotoi*. Only one *I. scapularis* nymph was co-infected with *B. burgdorferi* and *A. phagocytophilum*, and we did not detect *Ba. microti* in any of the ticks tested. At finer scales within both invaded and uninvaded sites, infection prevalence was positively related to coarse woody debris, distance to the nearest road, mouse abundance, and extent of understory cover within the forest fragment. We found a negative relationship between infection prevalence and both leaf litter and tick abundance. *Rosa multiflora* invasion and the additional factors positively influencing pathogen prevalence point to suitable habitat characteristics for small mammal and bird hosts that are competent pathogen reservoirs.

Invaded and uninvaded fragments represent two extremes of different, degraded habitat fragment types that can be separated by the presence/absence of *R. multiflora* invasion in our landscape. Uninvaded sites have deep litter layers, sparse understory, high densities of questing nymphs, and relatively low infection prevalence (mean = 0.079). Invaded sites have very little leaf litter, dense understory structure, fewer questing nymphs, and roughly double the infection prevalence (mean = 0.159). Our modeling results showed that the total understory cover in a forest fragment positively influences pathogen prevalence. Understory structure, which is provided almost exclusively by invasive plants, may aggregate immature ticks and infective hosts, resulting in increased pathogen prevalence among ticks in invaded forest fragments [19–21]. Because *B. burgdorferi* is not transmitted transovarially [44], infected free-living nymphs acquire the bacteria by feeding on an infected host during their larval stage. Similarly, potential pathogen hosts must acquire *B. burgdorferi* by being fed upon by an infected nymph. Therefore, both immature stages of ticks must interact with infected hosts to elevate pathogen prevalence among nymphs [45].

Understory structure facilitates interactions between immature ticks and competent *B. burgdorferi* reservoir hosts [22, 46, 47], but see [48]. White-footed mouse (*Peromyscus leucopus*) and breeding bird densities are positively correlated with understory structure [47, 49–51] [i.e. invasive plants, in our landscape (unpublished data)]. Within invaded forests, immature ticks are aggregated in stands of invasive shrubs [11, 20, 21]. We hypothesize that larval ticks in uninvaded sites derive a greater proportion of blood meals from larger-bodied hosts that are less-competent *B. burgdorferi* reservoirs [52, 53]. We expect that this is in contrast to larval tick blood meals in invaded sites, which we predict are composed of a greater proportion of small-bodied hosts that are positively affected by understory structure [46] and are competent *B. burgdorferi* reservoirs [53–55]. Future work should use blood meal analysis or identification of *ospC* types in *B. burgdorferi-*positive ticks to understand how non-native plant invasion affects the interaction between specific hosts and ticks, and the resulting implications for transmission of human-invasive *B. burgdorferi* strains [56–58].

An additional hypothesis to explain greater nymphal infection prevalence in invaded sites concerns tick overwinter survival. Invaded habitats lack the litter layer that comprises suitable off-host tick habitat [11, 16, 17]. Ticks depend on the high humidity microclimate within the litter to conserve moisture and to buffer themselves from environmental fluctuations [59]. However, saturated soils coupled with extremely low temperatures may also lead to decreased overwinter survival [60]. Recent studies show that ixodid ticks infected with *B. burgdorferi* have greater energy reserves and are more robust to desiccation [61–64]. Therefore, the harsh litter-free environment of invaded forests may exert stronger pressure against over-winter survival of uninfected ticks, thus increasing overall infection prevalence.

The negative relationships of nymphal infection prevalence with leaf litter and tick abundance raise questions about our understanding of Lyme disease ecology in over-browsed, mature forest fragments. Uninvaded, mature forest fragments that lack understory structure have greater litter volumes and questing tick abundance than invaded forests. We hypothesize that the lack of understory structure in uninvaded fragments shifts the composition of blood meal hosts toward reservoir-incompetent species such as white-tailed deer or other large-bodied hosts [53, 65]. Talleklint & Jaenson [66] also detected a negative relationship between tick density and infection prevalence at high tick densities (> 20 nymphs/m^2^), which they attributed to greater roe deer (*Capreolus capreolus*) densities. Elevated deer densities could account for both greater tick density and lesser infection prevalence if deer act as both reproductive hosts and the dominant blood meal source [66, 67]. The close proximity among our study sites suggests that deer do not account for differences in tick abundance; most sites are close enough to be within a single deer’s home range [68–70] (Additional file 1: Figure S1). However, deer may reduce infection prevalence by shifting blood meals away from reservoir competent hosts that do not find suitable understory cover in over-browsed, uninvaded fragments.

The importance of invasion, habitat, and landscape variables from our models suggest that understory structure and woody debris aggregate infectious hosts and larval ticks, increasing pathogen transmission. Coarse woody debris, total understory cover, distance to road, and white-footed mouse abundance, variables that directly or indirectly represent the distribution of reservoir hosts, were positively related to infection prevalence. Coarse woody debris provides cover, nest sites, movement corridors, and foraging opportunities for immature tick hosts such as white-footed mice, *Sorex* and *Blarina* shrews, and ground-foraging birds [71–75]. Shrews, in particular, are often overlooked in terms of their importance in the Lyme disease system, despite evidence that they can feed and infect more ticks than white-footed mice [76]. Outside of the Pacific Northwest and southern Appalachian regions of the USA, there is a dearth of studies on habitat associations of shrews [74]; in regions where shrews have been well studied, coarse woody debris appears to be an important habitat component [77–80]. Similarly, total understory cover represents the structure available to white-footed mice and shrub-nesting birds [47, 49, 50]. The importance of distance to road suggests that perhaps small mammals and birds avoid hard edges near roads in our landscape, or at least that larval ticks encounter infectious hosts farther from roads.

## Conclusions

Although nymphal infection prevalence was greater in invaded forests, acarological risk in terms of density of infected nymphs may be higher in uninvaded sites; the uninvaded sites examined in this study supported ~3 times as many questing nymphs compared to invaded sites [11]. Although uninvaded sites lack understory structure and therefore support lower densities of immature tick hosts, their relatively intact litter layers may allow nymphal ticks to survive longer [81] and quest more often [82], creating more opportunities to attach to humans than in invaded forests. Perhaps in uninvaded fragments, restoration of native understory structure [83] that promotes greater host diversity could reduce densities of questing infected nymphs.

## Additional files

Additional file 1: Figure S1. Reproduced without modifications from Adalsteinsson et al. Ecosphere. 2016;7(3):e01317 [11] under a Creative Commons license (CC BY 3.0). Map of study area in New Castle County, Delaware. Forest cover is green; agriculture is pale yellow; blue is water; and human development is white. Fragments designated as “rose-invaded” and “uninvaded” refer to the presence or absence of *Rosa multiflora* invasion (TIFF 7506 kb)

## References

[1] Bradley CA, Altizer S (2007). Urbanization and the ecology of wildlife diseases. Trends Ecol Evol.

[2] Allan BF, Keesing F, Ostfeld RS (2003). Effect of forest fragmentation on Lyme disease risk. Conserv Biol.

[3] LoGiudice K, Ostfeld RS, Schmidt KA, Keesing F. The ecology of infectious disease: effects of host diversity and community composition on Lyme disease risk. Proc Natl Acad Sci USA. 2003;100:567–71. 10.1073/pnas.0233733100 PMC141036 12525705

[4] LoGiudice K, Duerr ST, Newhouse MJ, Schmidt KA, Killilea ME, Ostfeld RS (2008). Impact of host community composition on Lyme disease risk. Ecology.

[5] Zolnik CP, Falco RC, Kolokotronis S-O, Daniels TJ (2015). No observed effect of landscape fragmentation on pathogen infection prevalence in blacklegged ticks (*Ixodes scapularis*) in the northeastern United States. PLoS One.

[6] Vellend M, Verheyen K, Flinn KM, Jacquemyn H, Kolb A, Van Calster H (2007). Homogenization of forest plant communities and weakening of species-environment relationships via agricultural land use. J Ecol.

[7] Huebner CD, Steinman J, Hutchinson TF, Ristau TE, Royo AA (2014). The distribution of a non-native (*Rosa multiflora*) and native (*Kalmia latifolia*) shrub in mature closed-canopy forests across soil fertility gradients. Plant Soil.

[8] Rogerson J, Globetti M, Hossler R, Moore E, Reynolds K, Hotton D, et al. Delaware Deer Management Plan. Delaware Department of Natural Resources and Environmental Control. 2010. http://www.dnrec.delaware.gov/fw/Hunting/Documents/Deer%20Plan%20-%20FINAL%2005212010.pdf. Accessed 20 Oct 2015.

[9] Rossell CR, Patch S, Salmons S (2007). Effects of deer browsing on native and non-native vegetation in a mixed oak-beech forest on the Atlantic coastal plain. Northeast Nat.

[10] Bernard MJ, Neatrour MA, McCay TS (2009). Influence of soil buffering capacity on earthworm growth, survival, and community composition in the western Adirondacks and Central New York. Northeast Nat.

[11] Adalsteinsson SA, D’Amico V, Shriver WG, Brisson D, Buler JJ (2016). Scale-dependent effects of nonnative plant invasion on host-seeking tick abundance. Ecosphere.

[12] Lawrence B, Fisk MC, Fahey TJ, Suárez ER (2003). Influence of nonnative earthworms on mycorrhizal colonization of sugar maple (*Acer saccharum)*. New Phytol.

[13] Suárez ER, Fahey TJ, Yavitt JB, Groffman PM, Bohlen PJ (2006). Patterns of litter disappearance in a northern hardwood forest invaded by exotic earthworms. Ecol Appl.

[14] Hale CM, Frelich LE, Reich PB (2006). Changes in hardwood forest understory plant communities in response to European earthworm invasions. Ecology.

[15] Nuzzo VA, Maerz JC, Blossey B (2009). Earthworm invasion as the driving force behind plant invasion and community change in northeastern north American forests. Conserv Biol.

[16] Schulze TL, Jordan RA, Hung RW (1995). Suppression of subadult *Ixodes scapularis* (Acari: Ixodidae) following removal of leaf litter. J Med Entomol.

[17] Burtis JC, Fahey TJ, Yavitt JB (2014). Impact of invasive earthworms on *Ixodes scapularis* and other litter-dwelling arthropods in hardwood forests, central New York state, USA. Appl Soil Ecol.

[18] Lubelczyk CB, Elias SP, Rand PW, Holman MS, Lacombe EH, Smith RP (2004). Habitat associations of *Ixodes scapularis* (Acari: Ixodidae) in Maine. Environ Entomol.

[19] Elias SP, Lubelczyk CB, Rand PW, Lacombe EH, Holman MS, Smith RP (2006). Deer browse resistant exotic-invasive understory: an indicator of elevated human risk of exposure to *Ixodes scapularis* (Acari: Ixodidae) in southern coastal Maine woodlands. J Med Entomol.

[20] Williams SC, Ward JS, Worthley TE, Stafford KC (2009). Managing Japanese barberry (Ranunculales: Berberidaceae) infestations reduces blacklegged tick (Acari: Ixodidae) abundance and infection prevalence with *Borrelia burgdorferi* (Spirochaetales: Spirochaetaceae). Environ Entomol.

[21] Allan BF, Dutra HP, Goessling LS, Barnett K, Chase JM, Marquis RJ, et al. Invasive honeysuckle eradication reduces tick-borne disease risk by altering host dynamics. Proc Natl Acad Sci USA. 2010;107:18523–7. 10.1073/pnas.1008362107 PMC2973004 20937859

[22] Prusinski MA, Chen H, Drobnack JM, Kogut SJ, Means RG, Howard JJ (2006). Habitat structure associated with *Borrelia burgdorferi* prevalence in small mammals in New York state. Environ Entomol.

[23] Kensinger BJ, Allan BF (2011). Efficacy of dry ice-baited traps for sampling *Amblyomma americanum* (Acari: Ixodidae) varies with life stage but not habitat. J Med Entomol.

[24] Falco RC, Fish D (1991). Horizontal movement of adult *Ixodes dammini* (Acari: Ixodidae) attracted to CO2-baited traps. J Med Entomol.

[25] Keirans JE, Durden LA (1998). Illustrated key to nymphs of the tick genus *Amblyomma* (Acari: Ixodidae) found in the United States. J Med Entomol.

[26] Keirans JE, Litwak TR (1989). Pictorial key to the adults of hard ticks, family Ixodidae (Ixodida: Ixodoidea), east of the Mississippi River. J Med Entomol.

[27] Durden LA, Keirans JE. Nymphs of the genus *Ixodes* (Acari: Ixodidae) of the United States: taxonomy, identification key, distribution, hosts, and medical/veterinary importance. Entomol Monogr. 1996;vol:1–50.

[28] Rega C. Impacts of soil calcium availability and non-native plant invasions on an urban forest bird community. University of Delaware 2012. http://udspace.udel.edu/handle/19716/11728. Accessed 2 Jun 2015.

[29] Nudds TD (1977). Quantifying the vegetative structure of wildlife cover. Wildl Soc Bull.

[30] Wolff JO (1985). The effects of density, food, and interspecific inference on home range size in *Peromyscus leucopus* and *Peromyscus maniculatus*. Can J Zool.

[31] State of Delaware, Office of Management and Budget, Delaware Geographic Committee. 2007 Delaware land use and land cover. 1st Edition. Dover, Delaware: State of Delaware, Office of Management and Budget, Delaware Geographic Data Committee. 2007. http://www.state.de.us/planning/info/lulcdata/2007_lulc.htm. Accessed 23 Jan 2014.

[32] Nicholson MC, Mather TN (1996). Methods for evaluating Lyme disease risks using geographic information systems and geospatial analysis. J Med Entomol.

[33] Bunnell JE, Price SD, Das A, Shields TM, Glass GE (2003). Geographic information systems and spatial analysis of adult *Ixodes scapularis* (Acari: Ixodidae) in the middle Atlantic region of the USA. J Med Entomol.

[34] Hojgaard A, Lukacik G, Piesman J (2014). Detection of *Borrelia burgdorferi*, *Anaplasma phagocytophilum* and *Babesia microti*, with two different multiplex PCR assays. Ticks Tick-Borne Dis.

[35] Bjornstad, ON. ncf: Spatial nonparametric covariance functions. 2016. https://cran.r-project.org/web/packages/ncf/ncf.pdf

[36] Development Core R, Team R, Language A. Environment for statistical computing. Vienna, Austria: R Foundation for Statistical. Computing. 2014; http://www.R-project.org

[37] Stan Development Team. Stan: A C++ Library for Probability and Sampling. 2015. http://mc-stan.org.

[38] Guo J, Lee D, Sakrejda K, Gabry J, Goodrich B, de Guzman J, et al. rstan: R Interface to Stan. 2016. https://cran.r-project.org/web/packages/rstan/.

[39] McElreath R. Rethinking: an R package for fitting and manipulating Bayesian models. 2016. https://github.com/rmcelreath/rethinking.

[40] McElreath R (2016). Statistical rethinking: a Bayesian course with examples in R and Stan.

[41] Watanabe S (2010). Asymptotic equivalence of Bayes cross-validation and widely applicable information criterion in singular learning theory. J Mach Learn Res.

[42] Gelman A, Hwang J, Vehtari A (2014). Understanding predictive information criteria for Bayesian models. Stat Comput.

[43] Yamashita T, Yamashita K, Kamimura RA (2007). Stepwise AIC method for variable selection in linear regression. Commun Stat Theory Methods.

[44] Rollend L, Fish D, Childs JE (2013). Transovarial transmission of *Borrelia* spirochetes by *Ixodes scapularis*: a summary of the literature and recent observations. Ticks Tick-Borne Dis..

[45] Buskirk JV, Ostfeld RS (1995). Controlling Lyme disease by modifying the density and species composition of tick hosts. Ecol Appl.

[46] Adler GH, Telford SR, Wilson ML, Spielman A (1992). Vegetation structure influences the burden of immature Ixodes dammini on its main host, *Peromyscus leucopus*. Parasitology.

[47] Willson MF, Comet TA (1996). Bird communities of northern forests: ecological correlates of diversity and abundance in the understory. Condor.

[48] Devevey G, Brisson D (2012). The effect of spatial heterogenity on the aggregation of ticks on white-footed mice. Parasitology.

[49] Adler GH, Wilson ML (1987). Demography of a habitat generalist, the white-footed mouse, in a heterogeneous environment. Ecology.

[50] Leston LFV, Rodewald AD (2006). Are urban forests ecological traps for understory birds? An examination using northern cardinals. Biol Conserv.

[51] Croci S, Butet A, Georges A, Aguejdad R, Clergeau P (2008). Small urban woodlands as biodiversity conservation hot-spot: a multi-taxon approach. Landsc Ecol.

[52] Cagnacci F, Bolzoni L, Rosà R, Carpi G, Hauffe HC, Valent M (2012). Effects of deer density on tick infestation of rodents and the hazard of tick-borne encephalitis. I: empirical assessment. Int J Parasitol.

[53] Barbour AG, Bunikis J, Fish D, Hanincová K (2015). Association between body size and reservoir competence of mammals bearing *Borrelia burgdorferi* at an endemic site in the northeastern United States. Parasit Vectors.

[54] Donahue JG, Piesman J, Spielman A (1987). Reservoir competence of white-footed mice for Lyme disease spirochetes. Am J Trop Med Hygeine..

[55] Ginsberg HS, Buckley PA, Balmforth MG, Zhioua E, Mitra S, Buckley FG (2005). Reservoir competence of native north American birds for the Lyme disease spirochete *Borrelia burgdorferi*. J Med Entomol.

[56] Brisson D, Dykhuizen DE (2004). ospC diversity in *Borrelia burgdorferi*: different hosts are different niches. Genetics.

[57] Önder Ö, Shao W, Kemps BD, Lam H, Brisson D (2013). Identifying sources of tick blood meals using unidentified tandem mass spectral libraries. Nat Commun.

[58] Vuong H, Canham CD, Fonseca DM, Brisson D, Morin PJ, Smouse PE (2014). Occurrence and transmission efficiencies of *Borrelia burgdorferi* ospC types in avian and mammalian wildlife. Infect Genet Evol.

[59] Stafford KC (1994). Survival of immature *Ixodes scapularis* (Acari: Ixodidae) at different relative humidities. J Med Entomol.

[60] Brunner JL, Killilea M, Ostfeld RS (2012). Overwintering survival of nymphal *Ixodes scapularis* (Acari: Ixodidae) under natural conditions. J Med Entomol.

[61] Naumov RL (2003). Longevity of forest and taiga ticks (Ixodidae) infected and non-infected with *Borrelia burgdorferi* groups. Parazitologiya.

[62] Herrmann C, Gern L (2010). Survival of *Ixodes ricinus* (Acari: Ixodidae) under challenging conditions of temperature and humidity is influenced by *Borrelia burgdorferi sensu lato* infection. J Med Entomol.

[63] Herrmann C, Gern L (2015). Search for blood or water is influenced by *Borrelia burgdorferi* in *Ixodes ricinus*. Parasit Vectors.

[64] Herrmann C, Voordouw MJ, Gern L (2013). *Ixodes ricinus* ticks infected with the causative agent of Lyme disease, *Borrelia burgdorferi sensu lato*, have higher energy reserves. Int J Parasitol.

[65] Telford SR, Mather TN, Moore SI, Wilson ML, Spielman A (1988). Incompetence of deer as reservoirs of the Lyme disease spirochete. Am J Trop Med Hygeine.

[66] Talleklint L, Jaenson TGT (1996). Relationship between *Ixodes ricinus* density and prevalence of infection with *Borrelia*-like spirochetes and density of infected ticks. J Med Entomol.

[67] Gray JS, Kahl O, Janetzki C, Stein J, Guy E (1995). The spatial distribution of *Borrelia burgdorferi*-infected *Ixodes ricinus* in the Connemara region of county Galway, Ireland. Exp Appl Acarol.

[68] Rhoads CL, Bowman JL, Eyler B (2010). Home range and movement rates of female exurban white-tailed deer. J Wildl Manag.

[69] Etter DR, Hollis KM, Deelen TRV, Ludwig DR, Chelsvig JE, Anchor CL (2002). Survival and movements of white-tailed deer in suburban Chicago, Illinois. J Wildl Manag.

[70] Grund MD, McAninch JB, Wiggers EP (2002). Seasonal movements and habitat use of female white-tailed deer associated with an urban park. J Wildl Manag.

[71] Roche BE, Schulte-Hostedde AI, Brooks RJ (1999). Route choice by deer mice (*Peromyscus maniculatus*): reducing the risk of auditory detection by predators. Am Midl Nat.

[72] Greenberg CH (2002). Response of white-footed mice (*Peromyscus leucopus*) to coarse woody debris and microsite use in southern Appalachian treefall gaps. For Ecol Manag.

[73] Lohr SM, Gauthreaux SA, Kilgo JC (2002). Importance of coarse woody debris to avian communities in loblolly pine forests. Conserv Biol.

[74] McCay TS, Komoroski MJ (2004). Demographic responses of shrews to removal of coarse woody debris in a managed pine forest. For Ecol Manag.

[75] Jones CG, Lindquist ES (2012). Utilization of woody debris by *Peromyscus leucopus* in a fragmented urban forest. Southeast Nat.

[76] Brisson D, Dykhuizen DE, Ostfeld RS (2008). Conspicuous impacts of inconspicuous hosts on the Lyme disease epidemic. Proc R Soc B Biol Sci.

[77] Carey AB, Johnson ML (1995). Small mammals in managed, naturally young, and old-growth forests. Ecol Appl.

[78] McCay TS, Laerm J, Menzel MA, Ford WM (1998). Methods used to survey shrews (Insectivora: Soricidae) and the importance of forest-floor structure. Brimleyana.

[79] Brannon MP (2000). Niche relationships of two syntopic species of shrews, *Sorex fumeus* and *S. cinereus*, in the southern Appalachian Mountains. J Mammal.

[80] Butts SR, McComb WC (2000). Associations of forest-floor vertebrates with coarse woody debris in managed forests of western Oregon. J Wildl Manag.

[81] Yuval B, Spielman A (1990). Duration and regulation of the developmental cycle of *Ixodes dammini* (Acari: Ixodidae). J Med Entomol.

[82] Randolph SE, Storey K (1999). Impact of microclimate on immature tick-rodent host interactions (Acari: Ixodidae): implications for parasite transmission. J Med Entomol.

[83] Morlando S, Schmidt SJ, LoGiudice K (2012). Reduction in Lyme disease risk as an economic benefit of habitat restoration. Restor Ecol.

