# Supplementary figures and images for: Multiflora rose invasion amplifies prevalence of Lyme disease pathogen, but not necessarily Lyme disease risk
Source: Parasit Vectors. 2018 Jan 23;11:54. doi: 10.1186/s13071-018-2623-0 (PMC5781316; doi:10.1186/s13071-018-2623-0)

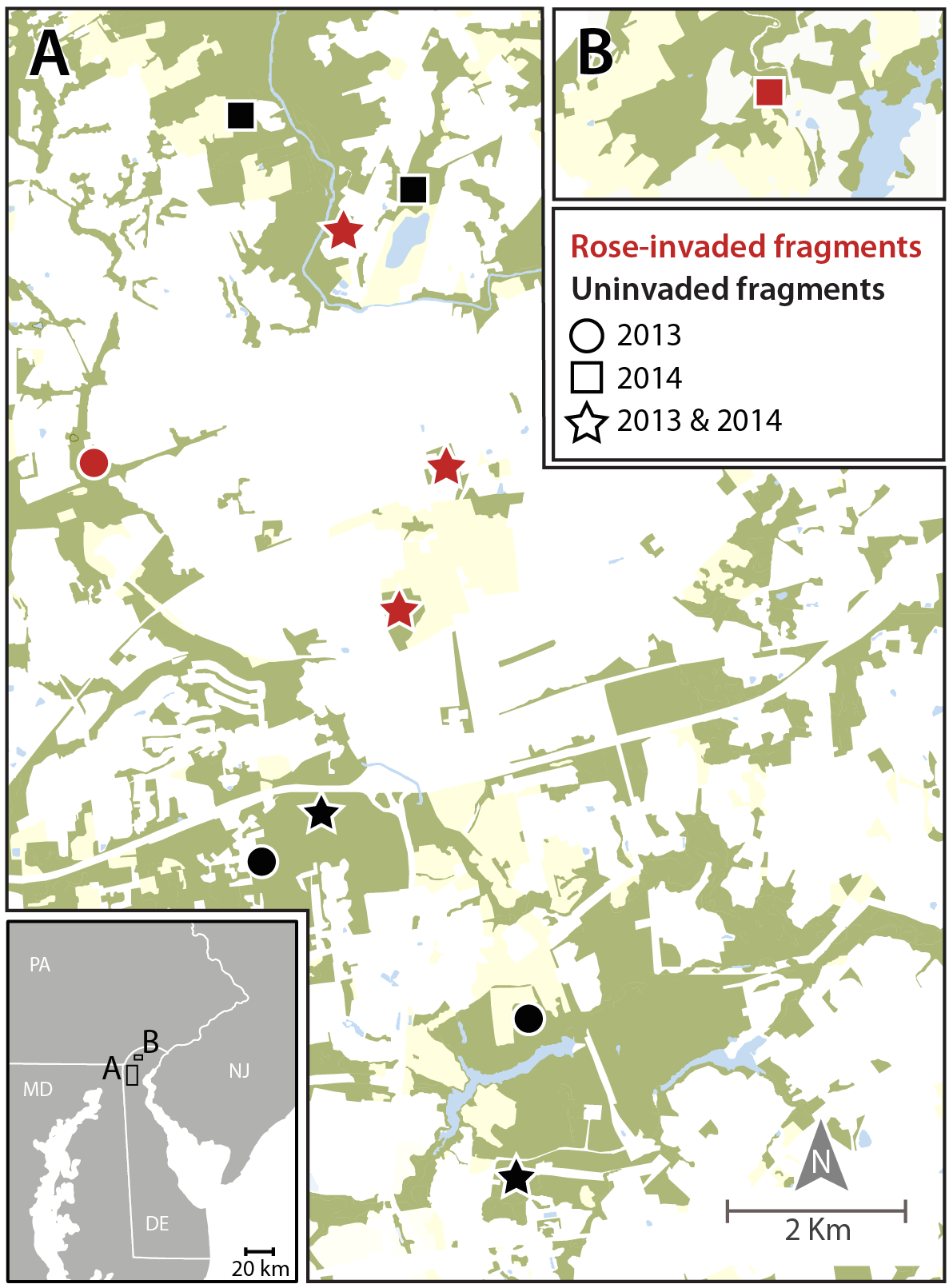

Supplement: Supplementary file 1 — Reproduced without modifications from Adalsteinsson et al. Ecosphere. 2016;7(3):e01317 [11] under a Creative Commons license (CC BY 3.0). Map of study area in New Castle County, Delaware. Forest cover is green; agriculture is pale yellow; blue is water; and human development is white. Fragments designated as “rose-invaded” and “uninvaded” refer to the presence or absence of Rosa multiflora invasion (TIFF 7506 kb) [file 13071_2018_2623_MOESM1_ESM.tif]
